# Biological age and lifestyle in the diagnosis of metabolic syndrome: the NHIS health screening data, 2014–2015

**DOI:** 10.1038/s41598-020-79256-4

**Published:** 2021-01-11

**Authors:** Chul-Young Bae, Meihua Piao, Miyoung Kim, Yoori Im, Sungkweon Kim, Donguk Kim, Junho Choi, Kyung Hee Cho

**Affiliations:** 1 MediAge Research Center, Sungnam, Republic of Korea; 2 Office of Hospital Information, Seoul National University Hospital, 101 Daehak-ro, Jongno-gu, Seoul, 03080 Republic of Korea; 3 Department of Big Data, National Health Insurance Corporation, Wonju, Republic of Korea; 4 Department of Family Medicine, National Health Insurance Service Ilsan Hospital, Goyang, Republic of Korea; 5 Research and Analysis Team, National Health Insurance Service Ilsan Hospital, Goyang, Republic of Korea

**Keywords:** Biomarkers, Endocrinology, Health care, Medical research

## Abstract

Metabolic syndrome (MS) is diagnosed using absolute criteria that do not consider age and sex, but most studies have shown that the prevalence of MS increases with age in both sexes. Thus, the evaluation of MS should consider sex and age. We aimed to develop a new index that considers the age and sex for evaluating an individual’s relative overall MS status. Data of 16,518,532 subjects (8,671,838 males and 7,846,694 females) who completed a validated health survey of the National Health Insurance Service of the Republic of Korea (2014‒2015) were analyzed to develop an MS-biological age model. Principal component score analysis using waist circumference, pulse pressure, fasting blood sugar, triglyceride levels, and high-density lipoprotein level, but not age, as independent variables were performed to derive an index of health status and biological age. In both sexes, the age according to the MS-biological age model increased with rising smoking and alcohol consumption habits and decreased with rising physical activity. Particularly, smoking and drinking affected females, whereas physical activity affected males. The MS-biological age model can be a supplementary tool for evaluating and managing MS, quantitatively measuring the effect of lifestyle changes on MS, and motivating patients to maintain a healthy lifestyle.

## Introduction

Metabolic syndrome (MS) is a global health problem involving a cluster of several cardiovascular risk factors^1^. The incidence of MS has been increasing worldwide, including in South Korea^2^. The Korea National Health and Nutrition Examination Survey reports that the prevalence of MS, assessed according to the modified National Cholesterol Education Program‒Adult Treatment Panel III (NCEP-ATP III) criteria, has increased from 24.9% in 1998 to 34.7% in 2012^3^. MS includes various combinations of insulin resistance, abdominal adiposity, dyslipidemia, and/or hypertension, and its prevalence increases with age. Moreover, MS is associated with a high risk of morbidity and mortality owing to cardiovascular disease (CVD) and type 2 diabetes^4^.

There is currently no standard definition for MS, causing confusion regarding whether they identify the same individuals or represent risk factor surrogates^5^. The NCEP-ATP III definition is the easiest to apply clinically and epidemiologically because it uses straightforward criteria that can be readily measured^6^. However, although metabolic risk factor clustering is consistent, the categorical diagnosis of MS is variable during adolescence^7^. Moreover, the conventional diagnosis of MS utilizes absolute criteria that does not consider the subject’s age and sex despite most studies showing that the prevalence of MS increases with age in both men and women^8–12^. Especially in women, the prevalence rapidly increases after menopause. Menopause is associated with the redistribution of body weight and weight gain in most women, and weight gain and obesity largely underlie the increased prevalence of MS in postmenopausal women^13^. This rise in prevalence is known to be due to the increase of chronic diseases such as central obesity, hypertension, diabetes, dyslipidemia, and deficiency in sex hormones with increasing age^14^.

Variables that show certain patterns according to age are called biomarkers of aging^15^. For example, waist circumstance is known as the most reliable biomarker by which we can diagnose central obesity and predict visceral fat amount, which increases with age^16^. Biological age (BA) estimated by the biomarkers of aging may serve as an indicator of identifying individuals at risk for age-related disorders, serving as a measure of relative fitness, and predicting disability in later life and mortality independent of chronological age^17^. Thus, MS diagnosis should consider biological age to overall evaluate and manage the health status and aging state in MS.

MS and its components, including dyslipidemia and hypertension, have been demonstrated to be common precursors of the development of type 2 diabetes and CVD and are risk factors for all-cause mortality^18^. Further, the risk of MS is known to be modified by diet, physical activity, smoking, drinking, and stress^19,20^. MS is associated with obesity and a sedentary lifestyle, both of which are modifiable, and thus, Misra et al. underlined the need for further efforts to promote a healthy lifestyle with increased physical activity to reduce obesity and the risk of MS^21^. Furthermore, individuals with MS should be identified at the early period therefore to attenuate their cardiovascular risk factors^22^. However, few studies have investigated approaches to quantify the effects of smoking, drinking, and physical activity in the risk of MS.

This study aimed to develop a new index that considers the age and sex for evaluating an individual’s relative overall MS status and to quantitatively assess the association between lifestyle factors, including alcohol drinking, smoking, and physical activity, and MS-BA. Towards this goal, we created an MS-biological age (MS-BA) model using data from the National Health Insurance Service (NHIS) of the Republic of Korea.

## Results

### General characteristics

In total, 16,518,532 subjects with an average age of 50.33 ± 14.3 years were evaluated. Of them, 8,671,838 were male (average age, 48.78 ± 14.16 years) and 7,846,694 were female (average age, 52.04 ± 14.26 years). The clinical parameters associated with MS are summarized in Table 1.

**Table 1:** General characteristics of the participants.

Parameters	Inclusion Criteria	Mean ± SD		
		Total (n = 16,518,532)	Male (n = 8,671,838)	Female (n = 7,846,694)
Age, years	> 20	50.33 ± 14.3	48.78 ± 14.16	52.04 ± 14.26
Waist circumference, cm	60–105	80.86 ± 8.84	84.17 ± 7.63	77.2 ± 8.63
**Blood pressure (mmHg)**				
Systolic	80–160	121.58 ± 13.45	123.57 ± 12.45	119.37 ± 14.16
Diastolic	50–100	75.55 ± 9.15	77.1 ± 8.78	73.82 ± 9.24
Mean arterial blood pressure		90.89 ± 9.83	92.59 ± 9.23	89.01 ± 10.12
Pulse pressure		46.03 ± 9.38	46.47 ± 8.96	45.55 ± 9.80
Fasting blood sugar (mmol/L)	2.78–7.77	5.33 ± 0.71	5.40 ± 0.72	5.25 ± 0.68
Triglyceride (mmol/L)	0.57–4.52	1.42 ± 0.73	1.56 ± 0.79	1.27 ± 0.63
High-density lipoprotein (mmol/L)	0.52–2.33	1.40 ± 0.32	1.33 ± 0.30	1.49 ± 0.32

Mean arterial blood pressure = (systolic blood pressure + 2 × diastolic blood pressure)/3; Pulse pressure = (systolic blood pressure − diastolic blood pressure). SI conversion factors: to convert fasting blood sugar to mmol/L, multiply by 0.0555; triglyceride to mmol/L, multiply by 0.0113; high-density lipoprotein to mmol/L, multiply by 0.0259.

*SD* standard deviation.

### MS-BA model

#### Correlation analysis and assessment of redundancy

The linear correlation between the diagnostic parameters of MS and age were analyzed. All parameters, except levels of triglycerides (TGs) and high-density lipoprotein cholesterol (HDL-C) in males and levels of HDL-C in females, were positively correlated with age (Table 2). The mean arterial blood pressure (MBP) and pulse pressure (PP) could be calculated based on systolic blood pressure (SBP) and diastolic blood pressure (DBP) and could have strong correlations with each other. Thus, we chose the parameter with the highest correlation with age as the blood pressure indicator (Table 2). PP reflected both SBP and DBP while excluding redundancy and collinearity between these two parameters with respect to the relationship with age. Therefore, although PP did not show the strongest correlation with age in females, PP was used as the blood pressure indicator in both sexes.

**Table 2:** The parameters influencing metabolic syndrome BA.

Parameters	Pearson correlation coefficients between age and parameters		Standardized scoring coefficients	
	Male	Female	Male	Female
Waist circumference, cm	0.092	0.356	0.42286	0.3722
Pulse pressure, mmHg	0.190	0.354	0.13926	0.25941
Fasting blood sugar, mmol/L	0.231	0.265	0.24737	0.28312
Triglyceride, mmol/L	− 0.020	0.238	0.43813	0.37087
High-density lipoprotein, mmol/L	− 0.037	− 0.221	− 0.40399	− 0.33814

PP (Pulse pressure) = systolic blood pressure − diastolic blood pressure.

All parameters are *P* < 0.001.

#### Principal component analysis

Based on the results of the correlation analysis, variables such as waist circumference (WC), PP, fasting blood sugar (FBS) level, TG level, and HDL-C level were selected as candidate biomarkers for the principal component analysis (PCA). First, factor analysis, including age, was performed to assess the association with age, and the principal components were found to have a significant positive correlation (0.389 in males, 0.700 in females) with age (Table 3). The proposed indicators, which correlated closely with age, were defined as principal components that reflected MS-BA. Second, we excluded age from the analysis to evaluate the influence of age and establish the correlation of the principal components with other biomarkers. This re-analysis confirmed the influence of age on the principal components. The principal components accounted for 32.54% of the total variance in males and 37.18% of that in females, with Eigenvalues of 1.627 and 1.859, respectively (Table 3).

**Table 3:** First principle components of the parameters for metabolic syndrome.

Parameters	First PCA loading			
	Male		Female	
	Age included	Age excluded	Age included	Age excluded
Age (years)	0.389	**–**	0.700	**–**
Waist circumference (cm)	0.666	0.688	0.681	0.692
Pulse pressure (mmHg)	0.321	0.227	0.535	0.482
Fasting blood sugar (mg/dL)	0.489	0.403	0.519	0.526
Triglyceride (mg/dL)	0.625	0.713	0.614	0.690
High-density lipoprotein (mg/dL)	− 0.599	− 0.657	− 0.558	− 0.629
Eigenvalue	1.685	1.627	2.198	1.859
Total variance (%)	28.08	32.54	36.63	37.18

Pulse pressure = systolic blood pressure – diastolic blood pressure. All parameters are *P* < 0.001.

*PCA* principal component analysis.

#### BA algorithm and correction of BA estimation equation

Principal component scores derived after excluding age were used as an index of health status and BA in MS. Regression analysis was performed using five candidate MS biomarkers (WC, FBS, TG, HDL-C, and PP) as independent variables. The equations developed after the process for calculating the biological age score (BAS) were as follows:

$$ \begin{aligned} {\text{BAS in males}} & = - {6}.{333} + 0.0{55} \times \left( {{\text{WC}}} \right) + 0.0{16} \times \left( {{\text{PP}}} \right) + 0.0{19} \times \left( {{\text{FBS}}} \right) + 0.00{6} \times \left( {{\text{TG}}} \right) - 0.0{34} \times \left( {{\text{HDL-C}}} \right) \\ {\text{BAS in females}} & = - {5}.{917} + 0.0{43} \times \left( {{\text{WC}}} \right) + 0.0{26} \times \left( {{\text{PP}}} \right) + 0.0{23} \times \left( {{\text{FBS}}} \right) + 0.00{7} \times \left( {{\text{TG}}} \right) - 0.0{27} \times \left( {{\text{HDL-C}}} \right) \\ \end{aligned} $$

It is difficult to explain BA to the general public as it is not expressed in terms of years. To overcome this disadvantage, BA was converted into years using the T-scale considering that the scores are distributed with a mean of 0 and a standard deviation (SD) of 1.0 as follows:

$$ \begin{aligned} {\text{BA in males}} & = ({\text{BAS in males}}\times {14}.{158}) + {48}.{779} \\ {\text{BA in females}} & = ({\text{BAS in females}}\times {14}.{261}) + {52}.0{4}0 \\ \end{aligned} $$

With the abovementioned relationship between age and BA, BA can be underestimated at the upper range of the equation and overestimated at the lower range. To reduce this systemic error, we used the following correction equation:

$$ \begin{aligned} & {\text{Corrected BA}} = {\text{BA}} + {\text{z}} \\ & {\text{z}} = \left( {{\text{yi}}{-}{\text{y}}} \right) \times \left( {{1}{-}{\text{b}}} \right) = \left( {{\text{age}}{-}{\text{mean age}}} \right) \times \left( {{1}{-}{\text{correlation with age and BA}}} \right) \\ & {\text{Corrected BA in males}} = - {82}.{688} + 0.{779} \times \left( {{\text{WC}}} \right) + 0.{227} \times \left( {{\text{PP}}} \right) + 0.{269} \times \left( {{\text{FBS}}} \right) \\ & \quad + 0.0{85} \times \left( {{\text{TG}}} \right){-}0.{481} \times \left( {{\text{HDL-C}}} \right) + 0.{857} \times \left( {{\text{age}}} \right) \\ & {\text{Corrected BA in females}} = - {6}0.{34}0 + 0.{613} \times \left( {{\text{WC}}} \right) + 0.{371} \times \left( {{\text{PP}}} \right) + 0.{328} \times \left( {{\text{FBS}}} \right) \\ & \quad + 0.{1}00 \times \left( {{\text{TG}}} \right){-}0.{385} \times \left( {{\text{HDL-C}}} \right) + 0.{538} \times \left( {{\text{age}}} \right) \\ \end{aligned} $$

**Figure 1:**
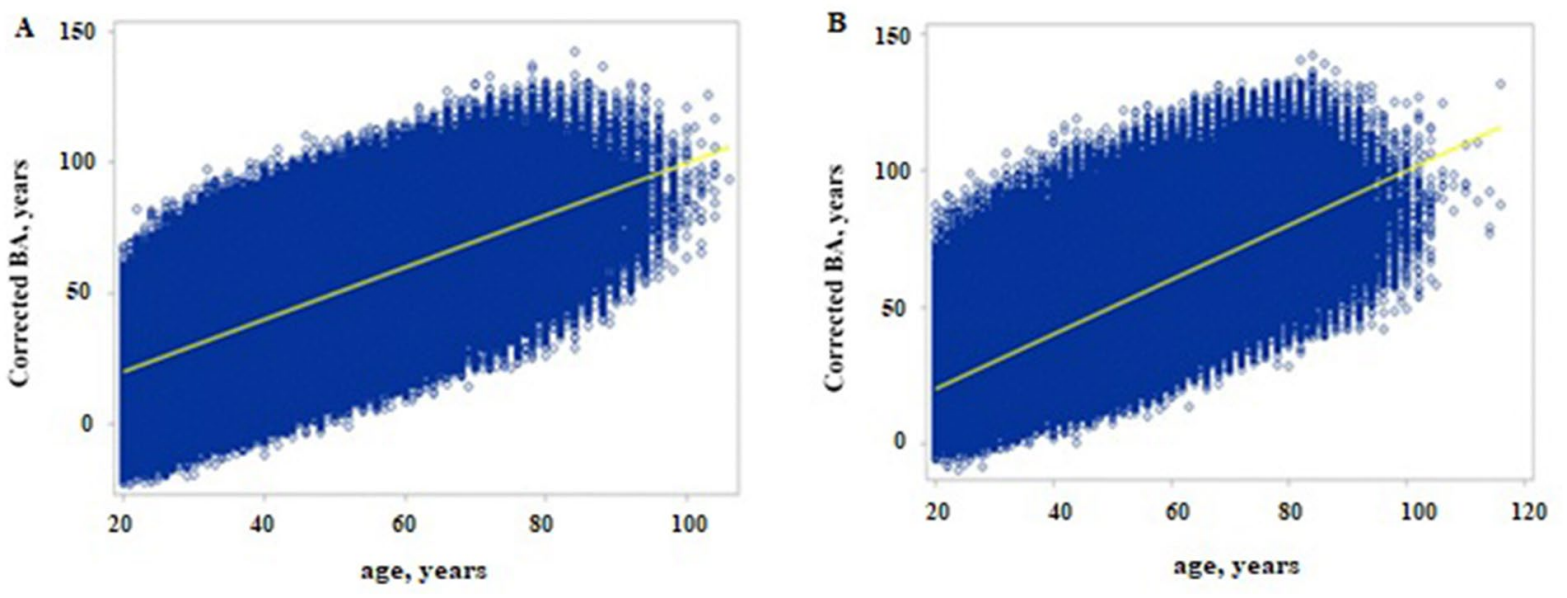
Relationship of MS BA in function of age for male (**A**), Female (**B**).

The correlation coefficients between the corrected BA (cBA) and age calculated from the abovementioned equation were 0.711 and 0.748 for the males and females, respectively. Through this correction, under- and overestimations of BA were avoided (Fig. 1).

#### Influencing parameters of MS-BA

The relative impact of the risk factors on MS-BA is shown in Table 2. The scoring coefficients obtained from PCA were standardized values without units of measurement. Larger numbers indicated a greater influence on MS-BA. The most influential parameter in males was TG (0.43813), while it was WC in females (0.3722).

### Clinical applications of the developed MS-BA model

To evaluate the possibility of the clinical application of the MS-BA estimation model, three patient categories were created: normal group, risk group (with 1‒2 risk indicators), and MS group. Additionally, the subjects were divided three groups by chronological age as the young group (20–< 40 years), middle group (40–< 60 years), and old group (≥ 60 years). Analysis of variance for the comparison of the mean differences between MS-BA and chronological age in the three groups showed significant differences (*P* < 0.001; Table 4). It should be noted that a positive difference between MS-BA and chronological age indicated a high risk, whereas a negative difference indicated a low risk.

**Table 4:** Clinical applications of the developed MS BA model.

Age group	Normal		Risk		Metabolic syndrome		*P* value
	N (%)	Mean ± SD	N (%)	Mean ± SD	N (%)	Mean ± SD	
**Male**							
TG (n = 8,671,838)	2,002,914 (100)	–12.64 ± 8.86	4,872,818 (100)	–0.78 ± 10.78	1,796,106 (100)	16.23 ± 10.11	< .0001
YG (n = 2,463,455)	823,782 (41.1)	–11.76 ± 8.66	1,287,877 (26.4)	0.94 ± 10.88	351,796 (19.6)	19.18 ± 9.97	< .0001
MG (n = 4,118,565)	857,424 (42.8)	–12.65 ± 8.83	2,338,587 (48.0)	–0.40 ± 10.73	922,554 (51.4)	16.45 ± 10.07	< .0001
OG (n = 2,089,818)	321,708 (16.1)	–14.84 ± 9.05	1,246,354 (25.6)	–3.28 ± 10.33	521,756 (29)	13.84 ± 9.68	< .0001
**Female**							
TG (n = 7,846,694)	2,677,507 (100)	–9.06 ± 8.07	3,889,567 (100)	0.77 ± 9.71	1,279,620 (100)	16.5 ± 10.56	< .0001
YG (n = 1,376,061)	840,140 (31.4)	–5.50 ± 7.47	480,365 (12.4)	6.64 ± 9.62	55,556 (4.3)	27.64 ± 9.94	< .0001
MG (n = 4,022,863)	1,480,041 (55.3)	–9.85 ± 7.58	2,042,509 (52.5)	1.71 ± 9.26	500,313 (39.1)	19.57 ± 9.82	< .0001
OG (n = 2,447,770)	357,326 (13.3)	–14.18 ± 7.72	1,366,693 (35.1)	–2.70 ± 9.05	723,751 (56.6)	13.53 ± 9.90	< .0001

Young group: 20.0 ≤ Age < 40.0 years, Middle group: 40.0 ≤ Age < 60.0 years, Old group: Age ≥ 60.0 years.

*TG* total group, *YG* young group, *MG* middle group, *OG* old group, *SD* standard deviation.

### Limitations of conventional MS diagnosis assessed using MS-BA

**Figure 2:**
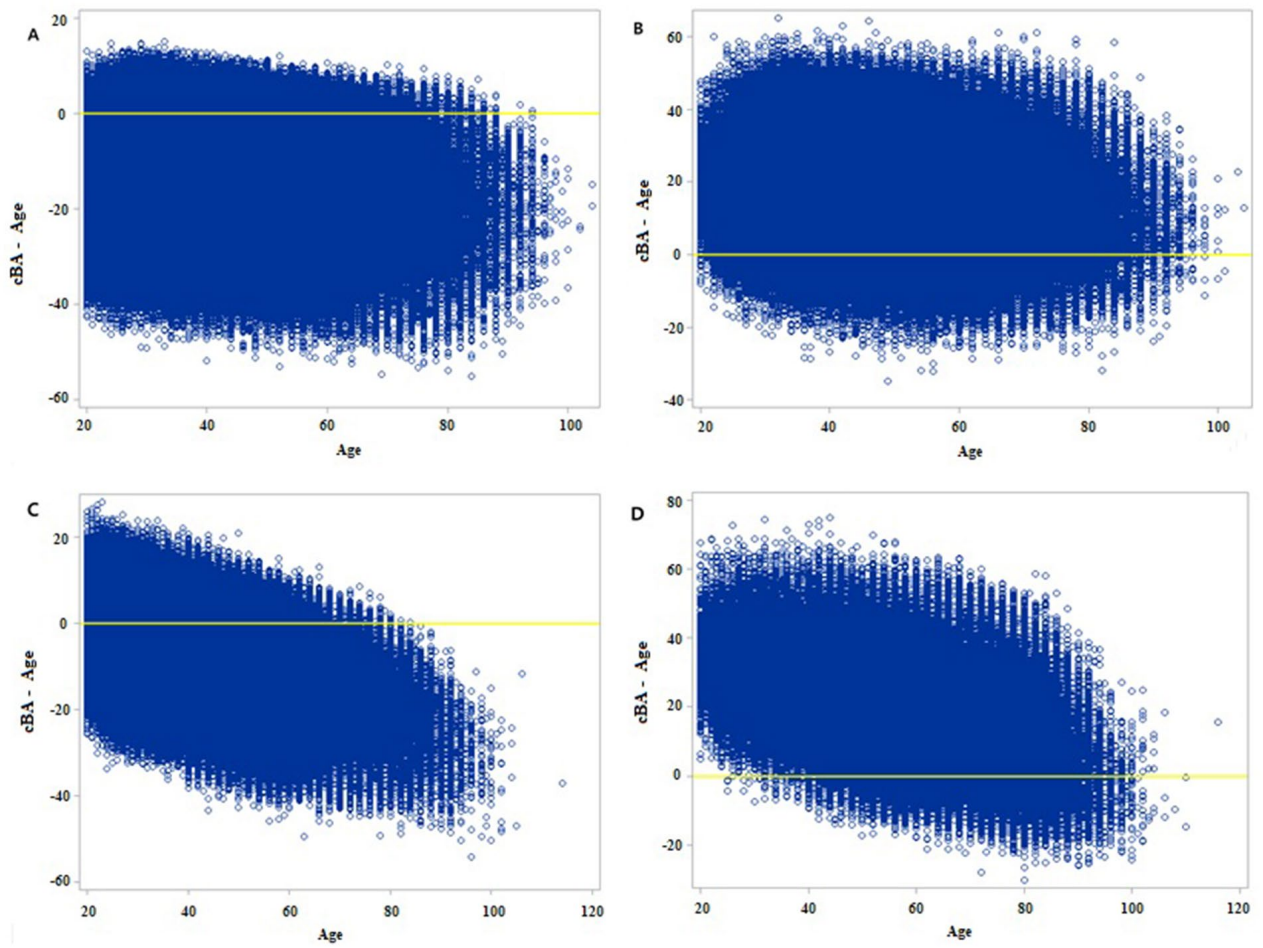
The scatter plot of discrepancy between conventional diagnosis. Y axis means the difference of MS BA and chronological age. X axis was chronological age. (**A**) normal male group, (**B**) MS male group, (**C**) normal female group, (**D**) MS female group.

In 6.49% of males and 12.9% of females who were assigned to the normal group based on conventional MS diagnosis, MS-BA was higher than chronological age (i.e., the difference exceeded zero). These participants, thus, appeared normal based on conventional diagnostic criteria, but were not normal when assessed using MS-BA. In contrast, 4.81% of males and 4.49% of females who were assigned to the MS group based on conventional diagnostic criteria had MS-BA lower than the chronological age (i.e., the difference was less than zero). These participants were conventionally diagnosed as having MS but were actually normal. If MS-BA were applied in parallel with the conventional diagnosis of MS, the MS status could be effectively identified and managed (Table 5; Fig. 2).

**Table 5:** The discrepancy between conventional diagnosis of metabolic syndrome and MS BA.

Conventional methods	cBA-age					
	Male			Female		
	Over zero N (%)	Under zero N (%)	Total	Over zero N (%)	Under zero N (%)	Total
Normal	130,029 (6.49%)	1,872,885 (93.51%)	2,002,914	345,460 (12.9%)	2,332,047 (87.1%)	2,677,507
Risk	2,378,695 (48.82%)	2,494,123 (51.18%)	4,872,818	2,059,727 (52.96%)	1,829,840 (47.04%)	3,889,567
Metabolic syndrome	1,709,784 (95.19%)	86,322 (4.81%)	1,796,106	1,222,160 (95.51%)	57,460 (4.49%)	1,279,620
Total	4,218,508 (48.65%)	4,453,330 (51.35%)	8671,838	3,627,347 (46.23%)	4,219,347 (53.77%)	7,846,694

All parameters are *P *< 0.001.

### Relationships between lifestyle factors and MS-BA

#### Smoking

Lifestyle-related biomarkers were selected based on the self-report questionnaire on health behavior administered during NHIS health screening examinations. Smoking status was divided into three categories: never smoker, former smoker, and current smoker. Smoking as a continuous variable was calculated as follows: pack/day $\times$ year. The mean and standard deviation of the differences between MS-BA and chronological age (cBA-age) were calculated according to the smoking categories. In both sexes, the differences in values increased as the amount of packs/day increased, indicating that smoking could increase MS-BA (Table 6). Smoking as a continuous variable was then included as an independent variable in the linear regression analysis. The equations were as follows:

$$ \begin{aligned} {\text{cBA-age in males}} & = - 0.{8763} + 0.0{8}0{62}\times ({\text{pack}}\times {\text{year}}),{\text{P}} < 0.00 \\ {\text{cBA-age in females}} & = - 0.0{668} + 0.{14135}\times ({\text{pack}}\times {\text{year}}),{\text{P}} < 0.00{1} \\ \end{aligned} $$

**Table 6:** The relationship between lifestyles and MS BA.

Lifestyles	Categories	cBA-age			
		Male		Female	
		N (%)	Mean ± SD	N (%)	Mean ± SD
Smoking	Never smoker	2,734,418 (31.5%)	–1.304 ± 13.604	7,413,754 (94.5%)	–0.109 ± 12.775
	Former smoker	2,641,443 (30.5%)	0.441 ± 13.662	163,979 (2.1%)	0.868 ± 13.228
	Current smoker	3,293,387 (38.0%)	0.737 ± 14.542	265,405 (3.4%)	1.945 ± 13.709
Alcohol consumption	Abstinent	701,381 (10.9%)	–0.605 ± 13.756	1,216,374 (37.4%)	–0.293 ± 12.587
	Low risk	5,125,490 (79.5%)	–0.0002 ± 14.038	1,845,045 (56.7%)	–0.855 ± 12.144
	Medium risk	425,377 (6.6%)	1.067 ± 14.650	144,624 (4.4%)	1.340 ± 12.494
	High risk	193,821 (3.0%)	1.906 ± 14.918	48,557 (1.5%)	2.377 ± 13.113
Physical activity	< 600METs	2,569,888 (29.6%)	0.654 ± 14.228	3,069,129 (39.1%)	0.571 ± 12.795
	≥ 600, < 3000METs	4,986,903 (57.5%)	0.023 ± 13.961	4,069,269 (51.9%)	–0.282 ± 12.530
	≥ 3000METs	1,115,047 (12.9%)	–1.591 ± 13.619	708,296 (9.0%)	–1.073 ± 12.494

All parameters are *P* < 0.001.

**Figure 3:**
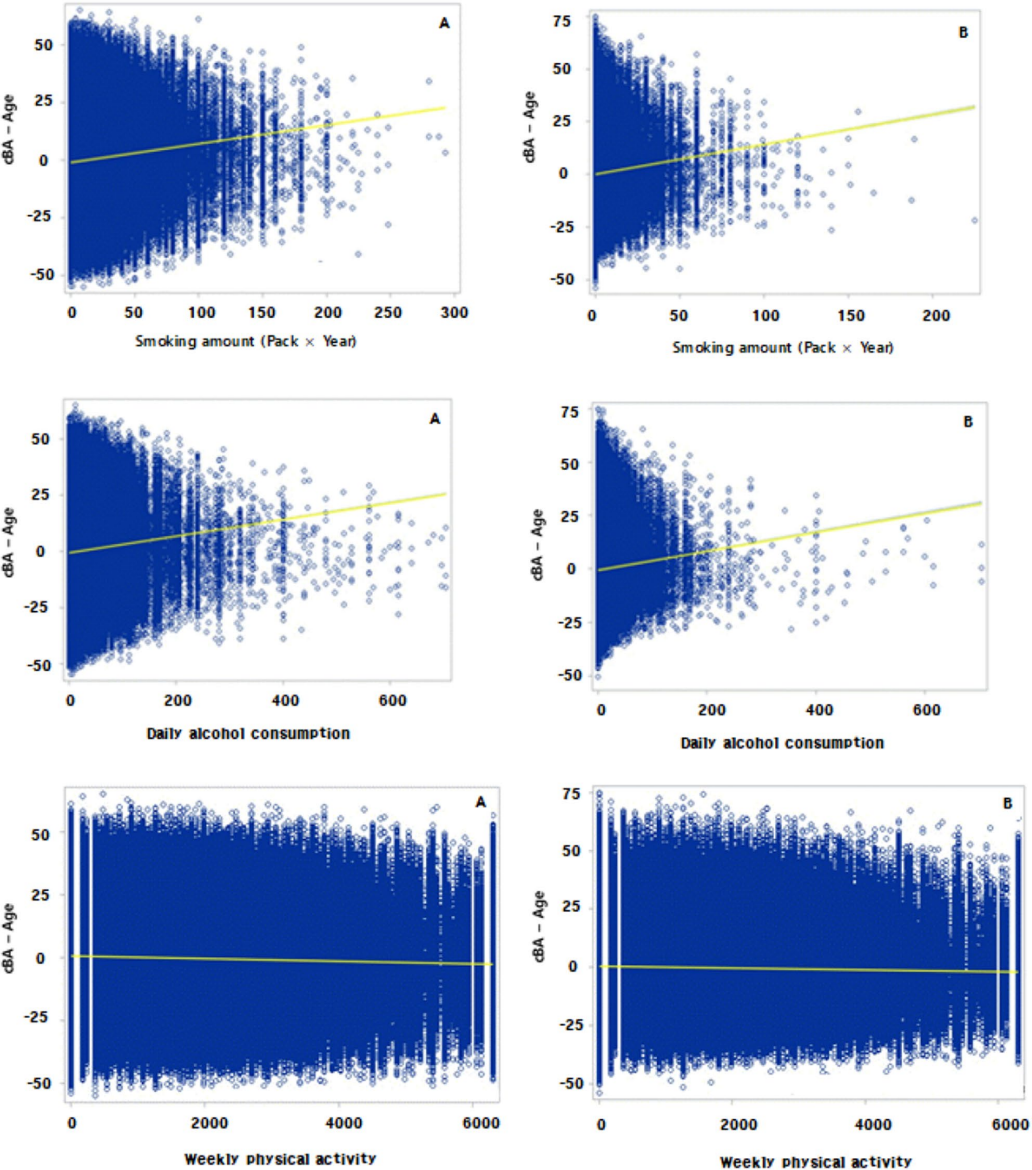
Relationships between lifestyle factors and MS BA (**A**: Male, **B**: Female).

The scatterplots are shown in Fig. 3. The results showed that the higher the amount of smoking, the worse was the MS-BA in both males and females.

#### Alcohol consumption

The daily alcohol consumption was calculated as the drinking amount (ml)/day $\times$ alcohol content (%) $\times$ 0.8 (alcohol gravity)/100, following the International Guide for Monitoring Alcohol Consumption and Related Harm of the World Health Organization (WHO). Alcohol consumption was categorized into four as abstinent, low risk, medium risk, and high risk. The mean and standard deviation of the differences between MS-BA and chronological age (cBA-Age) were calculated according to the alcohol consumption categories. We found that the greater the amount of alcohol consumed, the worse was the MS-BA in males. Similar tendencies were obtained among females, except in the low-risk group (Table 6). The daily alcohol consumption biomarker was included as an independent variable in the linear regression analysis. The equations were as follows:

$$ \begin{aligned} {\text{cBA-age in males}} & = - 0.{53628} + 0.0{37}0{1} \times \left( {\text{1 day alcohol amount}} \right),{\text{P}} < 0.00{1} \\ {\text{cBA-age in females}} & = - 0.{5}0{761} + 0.0{4419} \times \left( {\text{1 day alcohol amount}} \right),{\text{P}} < 0.00{1} \\ \end{aligned} $$

The scatterplots are shown in Fig. 3. The results showed that the greater the amount of alcohol consumed, the worse was the MS-BA in both males and females.

#### Physical activity

Physical activity was defined the sum of the physical activity levels for 1 week based on the WHO International Physical Activity Questionnaire (IPAQ) standard, according to which physical activity was divided into three categories: low level (< 600 MET*MIN), medium level (600‒3000 MET*MIN), and high level (> 3000 MET*MIN). The mean and standard deviation of the differences between MS-BA and chronological age (cBA-Age) were calculated for each of the physical activity categories. The more the physical activity, the greater the difference in MS-BA and chronological age in both males and females. This indicated that as the physical activity increased, the MS-BA decreased (Table 6).

The sum of 1 week of physical activity as a continuous variable was included as an independent variable in the linear regression analysis. The equations were as follows:

$$ \begin{aligned} {\text{cBA-Age in Males}} & = 0.{78723}{-}0.000{5245} \times \left( {{1} - {\text{week physical activity}}} \right),{\text{ P}} < \, 0.00{1} \\ {\text{cBA-Age in Females}} & = 0.{47183} - 0.000{4}0{39} \times \left( {{1} - {\text{week physical activity}}} \right),{\text{ P}} < \, 0.00{1} \\ \end{aligned} $$

The scatterplots are shown in Fig. 3. The results showed that the more the physical activity, the lesser the MS-BA in both males and females.

### Multiple regression analysis of lifestyle factors and MS-BA

The amounts of smoking, daily alcohol consumption, and 1 week’s physical activity as continuous data were included as independent variables in the multiple linear regression analysis. The equations were as follows:

$$\begin{aligned} {\text{cBA-age in males}} & = - 0.{419} + 0.0{25} \times ({\text{1 day's alcohol amount}}) - 0.000{512} \times \left( {{\text{1 week's physical activity}}} \right) \\ & \quad + 0.0{76} \times ({\text{smoking amount}}),{\text{P}} < 0.00{1} \\ {\text{cBA-age in females}} & = - 0.0{25} + 0.0{37} \times ({\text{1 day's alcohol amount}}) - 0.000{4123} \times \left( {{\text{1 week's physical activity}}} \right) \\ & \quad + 0.{132} \times ({\text{smoking amount}}),{\text{P}} < 0.00{1} \\ \end{aligned}$$

Compared with the results of the simple linear regression analysis, those of the multiple regression analysis showed that each of these independent variables had similar values and directions. As the smoking and drinking increased and physical activity decreased, the MS-BA increased in both males and females. In addition, the amount of smoking and drinking had a more profound impact in females more than males, whereas the amount of physical activity had greater effects in males than in females. These results allowed us to quantify the influence of smoking, drinking, and physical activity on MS-BA.

## Discussion

In this nationally representative longitudinal study, we demonstrated that an MS-BA model may be used as a supplementary tool to the conventional MS diagnostic criteria to more accurately evaluate and manage MS. Moreover, we found that in both sexes, the MS-BAs decreased as the amount of smoking and drinking increased and the amount of physical activity decreased. To the best of our knowledge, this is the first study to quantitatively measure the effects of lifestyle on the MS-BA in a large population.

Aging is characterized by a time-related decline in physiological functions, and the rate of aging differ among individuals owing to the variability of diseases^23^. However, chronological age is simply explained by the flow of time and has limitations when used to evaluate an individuals’ physiological function, health, and aging status^24^. The concept of BA has been widely investigated since the 1970s, and it has been proposed to quantify and digitize the aging state based on the age-related characteristic changes in physical and physiological functions^25^. There has been a growing interest in utilizing BA in chronic health management to compensate for the limitations of the binary structure of disease diagnosis^26–28^. BA is generally derived from a combination of biomarkers and represents an individual’s overall health and aging state in comparison with those of the same sex and age^25,29^. It has previously been reported that BA can be easily used as a continuous index to monitor health and aging status and can also be communicated easily to healthcare consumers^30–32^.

MS is diagnosed when three or more of the five parameters are outside of the reference range. However, the conventional diagnostic method has limitations in that it cannot reflect the current status of MS correctly with respect to an individual’s age and sex. For example, during the management of MS, some parameters may be well-controlled, while others could worsen. Thus, there is some difficulty in assessing whether the status of MS has really improved after lifestyle modification.

Numerous studies have assessed the effects of changes in MS components, such as serum glucose level, cholesterol level, blood pressure, and WC. The elevation of each of these components has been established to be associated with a higher MS risk. However, few studies have considered the individual association of these factors with MS-BA. In this study, WC, TG levels, and HDL-C level had high impact scores on MS-BA, and these scores were higher for males than for females. Meanwhile, PP and FBS had relatively lower scores than the other parameters, and the scores were lower in males than in females. Using the MS-BA index, which is a continuous variable, enables a closer assessment of the relationship between these parameters and MS. Further, their effects on the normal aging process, disease progression, and intervention outcome are measured and evaluated more objectively. In healthcare, indicators of outcome measures should be more relevant and sensitive. In this context, MS-BA can be utilized as a novel evaluation and management index in the assessment of the overall state of MS.

The present study demonstrated methods of quantitatively measuring the effects of lifestyle factors, such as smoking, drinking, and physical activity, on MS-BA. As continuous variables, we calculated the amount of smoking per year, alcohol consumption per day, and physical activity per week from the self-reported questionnaire on health behavior administered during the NHIS health screening examinations. We constructed linear regression equations with lifestyle behaviors and MS-BA. Therefore, our approach makes it possible to quantitatively measure the effects of changes in lifestyle behavior on MS-BA.

Our results support those of previous studies that found an influence of smoking on the development of visible signs of aging^20^. Smoking may increase sympathetic activity and circulating cortisol, catecholamine, vasopressin, and growth hormone levels. Therefore, it has been considered to play a causal role in the development of MS^33^. Additionally, current smokers, particularly those with excessive consumption, have a higher risk of MS^34,35^. Moreover, the relationship of smoking and alcohol consumption with the development of MS was found to be sex specific^36^. Similarly, we found that smoking increased the MS-BA and that the amount of smoking had a more profound impact on females than on males.

A previous meta-analysis showed that heavy alcohol consumption might be associated with a higher risk of MS; accordingly, very light alcohol consumption seemed to be associated with a lower risk of MS^37^. Further, this risk was greater in men than in women. In contrast, we found that alcohol consumption had a greater impact on the risk of MS in females than in males. Regular physical activity can yield physiological improvements that in turn reduces the rate of aging. A systematic review showed that the BA increased with decreased physical activity, irrespective of the sex^38^. Further, active middle-aged men who followed a regular endurance exercise program have a, on average, 4.7 years younger BA than their chronological ages^39^. However, it should be noted that an adequate amount of exercise is required to obtain the beneficial effects of exercise on cardiorespiratory function^40^. In agreement with these findings, we found that subjects with a high level of physical activity (> 3000 MET*MIN) had the lowest value of cBA-age in both sexes, which indicated a younger MS-BA. Physical activity could exert its protective effects against MS by improving plasma lipid levels, particularly through increases in HDL-C levels and decreases in TG levels. In addition, physical activity has been shown to lower blood pressure, improve glucose tolerance and insulin sensitivity, and lower the risk of type 2 diabetes^19^.

In conclusion, the MS-BA model accurately reflects the MS status relative to the individual’s age and sex. Thus, it can be a supplementary tool for evaluating and managing MS, quantitatively measuring the effect of lifestyle changes on MS, and motivating patients to maintain a healthy lifestyle*.* We hope that the estimation of MS-BA can facilitate the evaluation of the influence of age and sex on the MS status.

## Methods

### Study design and population

MS involves a clustering of abdominal obesity, elevated blood pressure, low serum HDL-C levels, elevated serum TG levels, and impaired FBS^41^. The modified NCEP-ATP III diagnostic criteria for MS^42^, developed by the American Heart Association/National Heart, Lung, and Blood Institute, stipulates that MS diagnosis requires meeting three of the following five criteria: central obesity (WC: males ≥ 90 cm, females ≥ 85 cm), high blood pressure (SBP/DBP ≥ 130/85 mmHg or medication intake), high TG levels (≥ 150 mg/dL or medication intake), low HDL-C levels (males < 40 mg/dL, females < 50 mg/dL, or medication intake), and fasting hyperglycemia (≥ 100 mg/dL or medication intake)^43^.

This study used the Medical and Health Examination database of the National Health Insurance Service-Health Screening Cohort (NHIS-HEALS). Data of 21,317,002 subjects who participated in the NHIS health screening examinations from 2014 to 2015 were collected from the NHIS-HEALS. Among them, 4,800,000 subjects were excluded due to the inclusion criteria listed in the Table 1.

This study was approved and exempted from review by the institutional review board of the NHIS Ilsan Hospital (NHIMC 2018–01-009) owing to the use of anonymized data.

### Data source

The NHIS of Korea was launched as a single insurance system and adopted as a compulsory social insurance program covering the whole population living in the country. Furthermore, the NHIS provides biannual health screening examinations for all citizens aged ≥ 40 years. These include a self-report questionnaire on health behavior; measurements of height, weight, and blood pressure; and urine and blood test results. The NHIS-HEALS provides cohort data of participants who undergo health screening examinations for research purposes^44^. It has been used in several epidemiological studies, and its validity is described in detail elsewhere^45,46^.

### Measures and definitions

Six biomarkers were selected: WC, SBP, and DBP as physical measures and FBS, TG, and HDL-C levels as blood test parameters. Additionally, lifestyle-related biomarkers were selected using the self-reported questionnaire on health behavior. Smoking was divided into three categories: never smoker, former smoker, and current smoker. Smoking amount as a continuous variable was calculated as follows: pack/day $\times$ year. Daily alcohol consumption was calculated as the drinking amount (ml)/day $\times$ alcohol content (%) $\times$ 0.8 (alcohol gravity)/100 based on the International Guide for Monitoring Alcohol Consumption and Related Harm. It was divided into four categories: abstinent, low risk, medium risk, and high risk. Physical activity was defined as the sum of physical activity levels for 1 week based on the WHO IPAQ standard. It was divided into three categories: low level (< 600 MET*MIN), medium level (600‒3000 MET*MIN), and high level (> 3000 MET*MIN)^47^.

### Statistical analysis

#### Correlation analysis and assessment of redundancy

MS diagnostic parameters, namely, WC, SBP, DBP, FBS level, TG levels, and HDL-C level, were used to develop the MS-BA model. First, linear correlation analysis of age and measured parameters was performed. Redundancy of the parameters was suspected based on the high level of correlation observed between the individual parameters in the correlation analysis. The correlation with age was assessed after calculating MBP and PP based on SBP and DBP to avoid redundancy, and both MBP and PP parameters were then used in model development^26^.

#### Principal component analysis

PCA was used to estimate MS-BA. First, PCA was performed using age and the five MS diagnostic parameters as variables. Among these factors, the factor with the highest Eigenvalue, which is the sum of the total variance of the parameter, was considered the principal component. We confirmed the changes in the factor loading value, which indicated the correlation between the factors and variables of PCA, after the exclusion of age.

#### Construction of BA

BAS was developed using the first principal component obtained from PCA of the selected biomarkers. Individual BAS was transformed into terms of years (BA) using the T-scale (transformation from a standard score to T-score) with consideration that these scores were distributed with a mean of 0 and an SD of 1.0. The formula for converting BAS to BA is as follows:

$$ {\text{BA}} = \left( {{\text{BAS}} \times {\text{standard deviation of CA}}} \right) + {\text{mean of CA}}. $$

#### Correction of BA estimation equation

Calculation of BA using the abovementioned formula underestimated the means for BA at the upper end of the regression and overestimated it at the lower end. To correct this systemic error, cBA was calculated using the following correction method:

$$ {\text{Corrected BA}} = {\text{BA}} + {\text{z}}. $$

The z value is as follows. z = (y_i_ − y) × (1 − *b*), where “y_i_” is the chronological age of an individual, “y” is the average chronological age of all samples, and *b* is the coefficient of simple linear regression, which expresses the relationship between BA and chronological age.

#### Multiple linear regression analysis between lifestyle factors and MS-BA

Multiple regression analysis was used to identify the relationship between age and lifestyle factors such as smoking, drinking, and physical activity.

All statistical analyses were performed using SAS version 9.4 (SAS Institute, Cary, NC, USA. https://www.sas.com/en_us/home.html). A two-sided *P* value of < 0.05 was considered statistically significant.

## Data Availability

The datasets generated during the current study are available from the corresponding author on reasonable request.
